# Supplementary material for: Content-rich biological network constructed by mining PubMed abstracts
Source: BMC Bioinformatics. 2004 Oct 8;5:147. doi: 10.1186/1471-2105-5-147 (PMC528731; doi:10.1186/1471-2105-5-147)
Supplement: Additional File 2 — The original results of the above study (non-essential files are deleted to keep the file size under the limit set by BMC bioinformatics). [file 1471-2105-5-147-S2.bz2 › chilibotAdditionalFile2/dip50/3ID9194558E4/html/TP53_BCL2.html]

 


 **TP53** and **BCL2** 
  
Found 77 abstracts in PubMed, retrieved 50.  
 

 What does Google say? 
 PDF only 
| .edu only 

---

**Interactive relationship** (e.g. stimulation, inhibition, etc)

**Neutral relationship**- All nine low labelling  **bcl2**  cases were  **TP53**  non reactive.  Ref: 10064395 Histopathology, 1999
- all six  **Tp53**  reactive cases were  **bcl2**  100% reactive.  Ref: 10064395 Histopathology, 1999

**Non-interactive relationship** (e.g. studied together, co-existance, homology, etc.)

- No association between BCL6 status and expression of  **BCL2** , Ki 67 or  **TP53**  was found.  Ref: 11743658 Int J Oncol, 2002
- **TP53**  mutation, p21 and BCL 2  [ **BCL2** ]  expression were not associated with increased rates of progression and death.  Ref: 11720475 Br J Cancer, 2001
- Using molecular and immunohistochemical analyses we examined  **TP53**  and its downstream genes p21, BAX and BCL 2  [ **BCL2** ]  in ovarian tumour tissues and have evaluated the results in relation to clinico pathological parameters, clinical outcome and response to platinum based chemotherapy.  Ref: 11720475 Br J Cancer, 2001
- Apoptosis was evaluated by TUNEL technique and the presence of EBV latent membrane protein 1 as well as expression of Bcl 2  [ **BCL2** ] , tumor suppressor p53  [ **TP53** ] , p21WAF1, MDM 2, Rb1, PCNA, p27KIP1 and caspase 3, was detected immunocytochemically on paraffin embedded lymph node specimens obtained at diagnosis.  Ref: 10938405 Int J Oncol, 2000
- Immunohistochemistry was performed for the mismatch repair proteins hMLH1 and hMSH2, tumor suppressor p53  [ **TP53** ] , apoptosis inhibitor bcl 2  [ **BCL2** ] , cell cycle proteins p21 WAF1 CIP1, and p27 and the proliferation markers Ki 67 and topoisomerase II.  Ref: 11150376 Hum Pathol, 2000
- Exposure of the lung to severe hyperoxia induces terminal transferase dUTP end labeling TUNEL indicative of DNA damage or apoptosis and increases expression of the tumor suppressor p53  [ **TP53** ]  and of members of the Bcl 2  [ **BCL2** ]  gene family.  Ref: 11140697 Lab Invest, 2000
- It was high in cases with mutant  **TP53**  and correlated with the total cellular level of the multidrug resistance associated protein P =.019 but not of bcl 2  [ **BCL2** ] , bax, or bcl x.  Ref: 11435310 Blood, 2001
- To elucidate the biochemical processes involved in the pathogenesis of B CLL and MCL, we analyzed the expression level of a set of genes that play central roles in apoptotic or cell proliferation pathways and of candidate genes from frequently altered genomic regions, namely ATM, BAX,  **BCL2** , CCND1, CCND3, CDK2, CDK4, CDKN1A, CDKN1B, E2F1, ETV5, MYC, RB1, SELL, TFDP2, TNFSF10, and  **TP53** .  Ref: 12036888 Blood, 2002
- The molecular events involved remain poorly defined but several genes are involved and mutations of  **tp53** , WAF1 CIP1, PTEN, bcl 2  [ **BCL2** ]  and c erbB 2 have been implicated.  Ref: 9822917 Histopathology, 1998
- PML bodies were found to localize specifically with the  **TP53**  locus in about 50% of Jurkat interphase nuclei, but never in proximity with the  **BCL2**  locus.  Ref: 12837275 Genomics, 2003
- We have found that whereas  **TP53** , BAK, ICH1 Short form, and DAD1 were expressed at constant levels, WAF1, PCNA, BAX were up regulated, ICH1 Long form, DNA LIGASE 1, and BCL 2  [ **BCL2** ]  were down regulated.  Ref: 10702800 Oncogene, 2000
- **TP53**  gene mutations, nuclear p53 accumulation, expression of Waf p21, Bcl 2  [ **BCL2** ] , and CD95 APO 1 Fas proteins are not prognostic factors in de novo glioblastoma multiforme.  Ref: 11519857 J Neurooncol, 2001
- Evaluation of clinical significance of  **TP53** , BCL 2  [ **BCL2** ] , BAX and MEK1 expression in 229 ovarian carcinomas treated with platinum based regimen.  Ref: 12644821 Br J Cancer, 2003
- In the present study, we have analyzed tumors of two age matched, equally treated groups of GBM patients with different postoperative time to tumor progression TTP, defined as short term for TTP of less than 6 months n = 54, and long term for TTP of more than 12 months n = 39 for alterations in apoptosis regulatory pathways Mutations of the  **TP53**  tumor suppressor gene and or nuclear accumulation of its gene product p53, expression of Waf p21, CD95 Apo1 Fas, and Bcl 2  [ **BCL2** ] .  Ref: 11519857 J Neurooncol, 2001
- **TP53**  status mutations, immunostaining, and loss of heterozygosity LOH, expression of c erbB 2, bcl 2  [ **BCL2** ] , and histological grading were correlated to the response to doxorubicin monotherapy 14 mg m2 administered weekly to 90 patients with locally advanced breast cancer.  Ref: 11289122 Cancer Res, 2001
- **TP53**  mutations, histological grade, and expression of bcl 2  [ **BCL2** ]  but not LOH or c erbB 2 expression all predicted for relapse free as well as breast cancer specific survival in univariate analysis Ps between < ; 0.0001 and 0.0155, but only tumor grade was found to be predictive in multivariate analysis P = 0.01 and P = 0.0007, respectively.  Ref: 11289122 Cancer Res, 2001
- Cytotoxicity because of substantial NO formation is established to initiate apoptosis, characterized by upregulation of the tumor suppressor p53  [ **TP53** ] , changes in the expression of pro and antiapoptotic Bcl 2  [ **BCL2** ]  family members, cytochrome c relocation, activation of caspases, and DNA fragmentation.  Ref: 12086396 J Environ Pathol Toxicol Oncol, 2002
- Many of these classes of molecules are oxidative responsive e.g., tumor suppressor p53  [ **TP53** ] , Bcl 2  [ **BCL2** ] , growth factors, immune derived proteins, and death inducing molecules and induce PCD by triggering a cascade of cysteine proteases and regulators e.g., caspases, death receptors.  Ref: 10682902 Crit Rev Oral Biol Med, 2000
- Another novel finding is that CR was negatively influenced by high BAX expression in all patients group P=0.047 and by  **BCL2**  expression in the  **TP53**  group P=0.05.  Ref: 12644821 Br J Cancer, 2003
- Cytotoxicity as a result of a substantial NO formation is established to initiate apoptosis, characterized by upregulation of the tumor suppressor p53  [ **TP53** ] , changes in the expression of pro and anti apoptotic Bcl 2  [ **BCL2** ]  family members, cytochrome c relocation, activation of caspases, chromatin condensation, and DNA fragmentation.  Ref: 10556974 Cell Death Differ, 1999
- Accumulation of wild type p53 protein was not related to bcl 2  [ **BCL2** ]  expression, a co expression of p53 and bcl 2  [ **BCL2** ]  was found at similar frequencies in tumours with or without  **TP53**  mutations 4 12 vs. 3 11.  Ref: 10606174 J Exp Clin Cancer Res, 1999
- We investigated a series of 14 patients with PCNSL to determine structural alterations of the INK4a ARF, MDM2, and  **TP53**  genes, the status of bcl 2  [ **BCL2** ]  and bcl 6 protein expression, and the clinical course of the patients i.e. their survival time after diagnosis.  Ref: 11804283 J Neurooncol, 2001
- To investigate the role of PML bodies in regulation of cell transformation and apoptosis related gene transcription, we employed the immuno FISH method to examine the relationship between PML bodies and the  **TP53**  and  **BCL2**  gene loci.  Ref: 12837275 Genomics, 2003
- However, initiation and continued progression toward cell death by apoptosis in cancer cells may be blocked by mutation of the tumor suppressor p53  [ **TP53** ]  or overexpression of members of the bcl 2  [ **BCL2** ]  family of proteins.  Ref: 12470246 Curr Med Chem, 2002
- We combined these approaches in a series of eight PCNSL cases, analyzing the chromosomal abnormalities using comparative genomic hybridization CGH, testing for Epstein Barr virus EBV involvement by in situ hybridization for EBER, assessing expression of p53, Bcl 2  [ **BCL2** ] , Bcl 6 and CD10 by means of immunohistochemistry, and screening for mutations of the  **TP53**  gene by DGGE.  Ref: 12802586 Virchows Arch< MedlineTA>Virchows Arch, 2002
- Cdk2 was found to function upstream of the tumor suppressor p53  [ **TP53** ] , transactivation of the death promoter Bax, alterations of mitochondrial permeability, Bcl 2  [ **BCL2** ] , caspase activation, and caspase dependent proteolytic cleavage of the retinoblastoma protein.  Ref: 10075979 J Exp Med, 1999
- Deregulated myc, bcl 2  [ **BCL2** ]  and or  **TP53**  gene expression is associated with non Hodgkin s B cell lymphomas B NHLs.  Ref: 10573140 Leuk Res, 1999
- Reduced expression of BAX is associated with poor prognosis in patients with epithelial ovarian cancer a multifactorial analysis of  **TP53** , p21, BAX and BCL 2  [ **BCL2** ] .  Ref: 11720475 Br J Cancer, 2001
